# Mr. James Sutherland

**Published:** 1921-02-05

**Authors:** 


### Mr. James Sutherland.

"THAT CHAP INTERESTED IN THE INFIRMARY."

By such a title was the late Mr. James Sutherland, of Melton Mowbray, known over a wide area. Although only fifty-eight years of age, he was one of the older type of hospital workers who "did things." He never spared himself in seeing that his plans were thoroughly carried out; and it would have delighted him to know that he was spoken of in the language of the above heading. Some years ago he became a patient in the Leicester Royal Infirmary, and from that time his personal interest began. It was due to him in bygone days that considerable sums of money were raised locally by means of Friendly Society parades—for he undertook the secretarial duties and infected all his colleagues with the desire to "beat last year's record." For his services in this direction he was made a life governor of the infirmary—a position of which he was genuinely proud.

His interests were many. A basket-maker by trade, he made his business help the infirmary by the loan of baskets to vegetable producers and to churches desiring to send surplus supplies to the institution, and he undertook to forward such produce to Leicester. He was one of the originators of the local branch of the Leicester Saturday Hospital Society and honorary secretary of the branch from its start. On every possible occasion he urged the duty of workers to contribute, and it was a great pride to him to see the local Society develop into a flourishing branch. His work came to a sudden end after addressing a meeting of the Waltham Friendly Society, but it was a death in harness, as he would have wished. The greatest sympathy is expressed with his wife and family in their loss.

